# Effect of 2-hydroxyethylammonium carboxylate protic ionic liquids on the solubility and cytotoxicity of indomethacin

**DOI:** 10.1186/s13065-024-01212-4

**Published:** 2024-06-03

**Authors:** Parisa Akbarzadeh Gondoghdi, Mohammad Khorsandi, Masumeh Mokhtarpour, Hemayat Shekaari, Hamed Hamishehkar

**Affiliations:** 1 Department of Physical Chemistry, University of Tabriz, Tabriz, Iran; 2 Drug Applied Research Center, Tabriz University of Medical Sciences, Tabriz, Iran; 3 Research Center for Bioscience and Biotechnology, University of Tabriz, Tabriz, Iran

**Keywords:** Solubility, Indomethacin, Protic ionic liquids, MTT assay, Cytotoxicity

## Abstract

Recently, there is a particular interest to utilize protic ionic liquids (PILs) in drug solubility. This study is exploring the effect of three protic ionic liquids (PILs) based on 2-hydroxyethylammonium carboxylate [2-hydroxyethylammonium acetate (MEAA), 2-hydroxyethylammonium lactate (MEAL), and 2-hydroxyethylammonium propionate (MEAP)] on the solubility of the very poorly soluble drug in water, indomethacin (IMC). The shake flask method was used to measure the experimental solubility of IMC at the different temperatures range (298.15–313.15) K. The results demonstrate significantly enhancment the solubility of IMC in PILs compared to pure water, with an approximate increase of 200 times. The experimental solubility data have been correlated using the empirical models which showed the performance as the order: Modified Apelblat–Jouyban–Acree > Van’t Hoff–Jouyban–Acree > Modified Apelblat equations and also the performance for the Wilson model indicated as the order (absolute relative deviation): 2-hydroxyethylammonium acetate (3.030) > 2-hydroxyethylammonium propionate (3.239) > 2-hydroxyethylammonium lactate (7.665). Then the thermodynamic dissolution properties were obtained by usage of Gibbs and Van’t Hoff equations to investigate the thermodynamic behavior of the IMC in the aqueous solution PILs. Eventually, the cytotoxicity of the co-solvents (PILs) under study was evaluated using a standard MTT assay. The results showed that the cell viability percentage increased in the following order: MEAA < MEAP < MEAL. These findings indicated that these PILs had low to moderate toxicity. It is noteworthy that the functional groups of the anions were not the only determinant factor of the cytotoxicity. Other factors encompassing concentration, exposure time, and cell line characteristics also had significant effects.

**Supplementary Information:**

The online version contains supplementary material available at 10.1186/s13065-024-01212-4.

## Introduction

Ionic liquids (ILs) are used recently as class of neoteric green solvents with potential applications in various fields of chemical and pharmaceutical industries. The ILs can be divided into two major groups based on the nature of their cations which could be called as the aprotic ionic liquids (AILs), with neutral cations, and protic ionic liquids (PILs), with protonated cations. The PILs exhibit distinct characteristics from AILs due to the presence of the acidic proton in their cations [1]. The presence of the acidic proton in the cation of PILs confers them several advantages over ILs, encompassing the ability to modify their physicochemical properties by changing the acidity, the enhancement of their catalytic activity by acting as Bronsted acids, and the improvement of their stability under acidic environments. Furthermore, the PILs typically (carboxylic anions and ammonium-based cations—specifically, tris(2-hydroxyethyl)ammonium, bis(2-hydroxyethyl)ammonium, and 2-hydroxyethylammonium) show non-flammability, low toxicity, and lower vapor pressure than volatile organic compounds (VOCs), making them appealing substitutes for VOCs in various applications in the pharmaceutical and chemical industries [2]. These PILs are attractive in chromatography based on their ability to solubilize a wide range of compounds, including hydrophilic and hydrophobic molecules. In biotechnology, the PILs provide stability and biocompatibility, making them applicable for enzyme catalysis and drug delivery applications [3]. Moreover, they can enhance the solubility and extraction of various substances, including pharmaceuticals, pigments, and heavy metals. Additionally, their ability to buffer pH and mimic the properties of water makes them useful for thermodynamic studies [4]. Furthermore, the utilization of green solvents has become widespread in various industries, as the medical field, due to their advantages over traditional organic solvents [5]. Recently, there has been a growing interest in ionic liquids specially protic ionic liquids as sustainable solvents in the scientific community, particularly in the field of green chemistry. In this regard, they are gaining attention as environmentally friendly alternatives to hazardous solvents, and the pharmaceutical industry is increasingly considering their application.

Particularly during pharmaceutical development, selecting the appropriate dosage of a drug is crucial to achieving the desired pharmacological effects. Various methods have been employed to address the challenges associated with achieving adequate drug solubility including pH adjustment, complexation, cyclodextrins, solid dispersions, and co-solvency [6]. Co-solvency, due to its convenience and cost-effectiveness, is widely used. It entails incorporating a small amount of a secondary solvent to increase the solubilizing power of the primary solvent [7]. However, selecting solvents for various pharmaceutical processes, including purification, chemical reactions, and drug dissolution, is a critical consideration. In the last decade, investigations based on the protic ionic liquids (PILs) and low-melting mixtures (LMMs) as potential alternatives to conventional organic solvents to address this issue have been developed [8]. These PILs adaptability by combining various cations and anions is one of their key advantages, expanding their applications in the chemical and pharmaceutical industries [9, 10].

On the other hand, the nonsteroidal anti-inflammatory medicine (NSAID) indomethacin (IMC) has been recognized for its analgesic, anti-inflammatory, and antipyretic properties. IMC has a wide range of medicinal applications and is frequently employed in the pharmaceutical industry to treat pain associated with menstrual cramps, postoperative pain, and other types of pain. On the flip side, the IMC is classified as a class II medication in the biopharmaceutics classification system (BCS) due to its poor water solubility (2.5 mg/mL to 4.0 mg/mL), leading to the need for the creation of more effective pharmaceutical formulations [11–14].

**Fig. 1:**
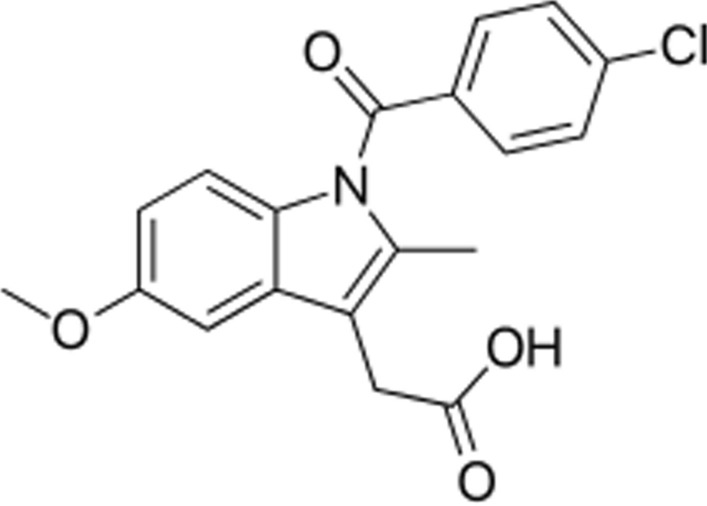
Indomethacin (IMC) molecular structure

This study extends the authors’ previous research on the utilization of environmentally friendly solvents, the protic ionic liquids (PILs) as co-solvents, to investigate the impact of PILs on the solubility of indomethacin (IMC) as a nonsteroidal anti-inflammatory drug (NSAID) with analgesic, anti-inflammatory, and antipyretic properties (Fig. 1) [15–19]. The study specifically concentrates on three newly developed PILs with 2-hydroxyethylammonium as the cation and various carboxylate as the anion, namely 2-hydroxyethylammonium propionate (MEAP), 2-hydroxyethylammonium lactate (MEAL), and 2-hydroxyethylammonium acetate (MEAA), to determinate their impact on the experimental solubility of the IMC at various concentrations of the PILs and different temperatures. Additionally, the experimental solubility data were correlated to several models, including empirical models (the Van’t Hoff–Jouyban–Acree model, Jouyban–Acree model, and the Jouyban–Acree model) and Wilson model as the local composition model [20, 21]. Furthermore, apparent thermodynamic properties of dissolution were calculated using the equations of Gibbs and Van’t Hoff to determine the thermodynamic behaviour of the IMC in aqueous systems containing PILs. This study contributes to the existing research on environmentally friendly solvents, offering valuable insights for the pharmaceutical industry to explore alternative solvents that are sustainable and environmentally benign.

## Materials and methods

### Materials

In this research, the materials utilized were sourced from reputable suppliers to ensure their quality and purity. The  chemicals were purchased from Sigma-Aldrich Co. and Merck, supplied  monoethanolamine (2-hydroxyethylammine), lactic acid, propionic acid, indomethacin and acetic acid, all of which had a mass fraction purity of more than 0.99. The doubly distilled deionized water was used to preparation of the solutions. The relevant information about the components used in the study, including their sources, CAS numbers, molar masses, purities, compounds structure were provided in Table 1.

**Table 1:** Information about the chemicals employed, CAS number, purity, and chemical structure

Chemical name	Provenance	CAS no.	Mass fraction (purity)	Structure
Indomethacin (IMC)	Merck	53-86-1	> 0.99	
Monoethanolamine (MEA)	Merck	141-43-5	> 0.99	
Propionic acid	Merck	79-09-4	> 0.99	
Acetic acid	Merck	64-19-7	> 0.99	
l-(+)-Lactic acid	Merck	79-33-4	88–92	

The suppliers were provided the purities of the used components

### Protic ionic liquid synthesis and characterization

The present study involved the synthesizing and pacificating of protic ionic liquids (PILs) using a neutralization method. The monoethanolamine (2-hydroxyethylamine) and carboxylic acids (lactic acid, acetic acid and propionic acid) were employed as starting materials to synthesize PILs, including 2-hydroxyethylammonium lactate, 2-hydroxyethylammonium acetate and 2-hydroxyethylammonium propionate. The synthesis process involved stirring the 2-hydroxyethylamine in a three-neck glass flask, then gradually adding carboxylic acids using a dropping funnel while stirring at room temperature. The resulting viscous liquid was purified using vacuum evaporation for 12 h at 343 K to eliminate any volatile impurities [22]. The purity of the PILs was analyzed using ^1^H NMR spectra and was found to be more than 97%. To determine the water contents of the synthesized ionic liquids, the Karl–Fisher titration technique (method TitroLine KF) was used (Table 2).

**Table 2:** Common properties of ionic liquids used in this work at 298.15 K and 866 hPa

PILs	*M*_*P*ILs_ (g mol^−1^)	CAS number	Purification method	Mass percent (purity)	Water content (ppm)	Analysis method
2-Hydroxyethylammonium propionate (MEAP)	135.16	90434-46-1	Rotary evaporator	> 97	209	H’NMR
2-Hydroxyethylammonium acetate (MEAA)	121.13	54300-24-2	Rotary evaporator	> 97	186	H’NMR
2-Hydroxyethylammonium lactate (MEAL)	151.16	68815-69-0	Rotary evaporator	> 97	235	H’NMR

Standard uncertainty for u*(T)* = 0.1 K and u*(P)* = 10 hPa

### Solubility measurement

**Fig. 2:**
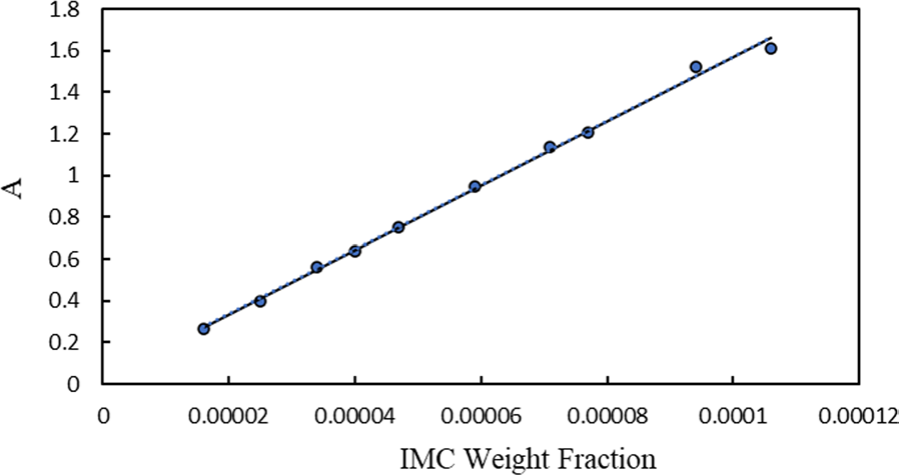
Calibration curve of indomethacin (IMC)

Before obtaining dissolution data, a calibration curve for Indomethacin (IMC) was established (Fig. 2). A double-beam T80 UV–vis spectrometer (Japan) and a mixture of ethanol and distilled deionized water were employed to dissolve a specific amount of IMC to generate the calibration curve [23]. Numerous methods were utilized to determine the experimental solubility data, encompassing the shake-flask method [24]. Aqueous binary mixtures containing different weight fractions of protic ionic liquids (PILs) were prepared using an analytical balance with a precision of 10^–4^ g (AW 220, GR220, Shimadzu, Japan) in order to determine the experimental solubility. Then, the excess amounts of IMC were added to glass vials holding a particular quantity of water and PILs, which were stirred and kept for 3 days in a water bath thermostat until equilibrium was reached. The temperature was adjusted with a precision of 0.01 K using an ED water bath thermostat (Julabo Co., Germany). After 3 days, the liquid and solid phases were separated employing a Hettich D-7200 centrifuge. At the next step, the liquid phase was appropriately diluted with an ethanol + water solution after filtering the saturated solutions through a 0.22 µm PTFE filter. The concentration of IMC in the solutions was measured by utilizing the calibration curve and a T80 UV–vis spectrometer (Japan) [24].

The mole fraction of IMC (x_1_) in the aqueous solutions containing PILs was determined, for the IMC + water, IMC + water combination, and IMC + PILs systems using the Eq. 1 [25, 26]:

1 $$x_{1} = \frac{{\frac{{w_{1} }}{{M_{1} }}}}{{\frac{{w_{1} }}{{M_{1} }} + \frac{{w_{2} }}{{M_{2} }} + \frac{{w_{3} }}{{M_{3} }}}},$$

where *W*_i_ and *M*_i_, respectively, indicate the weight fractions and molar mass weight fractions of each system component (i).

### Cell culture

Pastor Institute of Iran generously provided the human colon adenocarcinoma cell line HT29. The cell lines were cultured in RPMI’s medium supplemented with 10% fetal bovine serum (FBS) and Culture medium containing FBS. The cell cultures were incubated at 37 °C in a humidified environment of 5% carbon dioxide—CO_2_. A Nikon Eclipse 80i inverted microscope was employed to investigate cell morphology (Nikon, Tokyo, Japan).

### MTT assay

The colorimetric MTT assay was used to assess the cytotoxic activity of PILs (Twentyman and Luscombe 1987). HT29 cells were seeded in 96-well plates and allowed to conform for the first step. The cells were then incubated for 24 h at 37 °C in 5% CO_2_ in a total of six concentrations (3–150 µg mL^−1^) of the studied compounds diluted in RPMI medium (previously sterilized with a 0.22 m syringe filter). After 24 h of exposure, each well was filled with 50 µL of MTT solution (3 (4,5-dimethylthiazol-2-yl)-2,5-diphenyltetrazolium bromide (MTT) from Sigma-Aldrich) (2 mg. mL in PBS, pH 7.2). To dissolve the formazan crystals, the medium was substituted with 150 µL of dimethyl sulfoxide (DMSO) after 4 h of incubation.

The plate was shaking for around 1 h while being held out of direct sunlight. Cell viability was measured through the optical density of reduced MTT at 570–630 nm using a microplate reader (Synergy HT from BioTeK Instruments Inc., Winooski, VT, USA). The percentage of viable cells was calculated as the ratio between the absorbance of treated and control cells. Similarly, IC50 was estimated using a non-linear regression, logistic equation to determine the chemical concentration that causes a 50% reduction in cell viability [27].

### Solubility data correlation procedure

A crucial factor in the pharmaceutical industry could be experimental solubility measurement and the significance of thermodynamic models in correlating the solubility of pharmaceuticals in various solvents. In modeling, the clarity of the model parameters, which is obtained with the minimum number of experiments, gives the researcher the power to predict and correlate the solubility in other concentrations and conditions which is possible to calculate the most suitable concentration of the solvent for the formulation of the drug. By considering of the thermodynamic models application, they containing some wonderful advantages encompassing saving the time, decreasing the experimental costs, correlating and predicting the experimental data in the unusual experimental conditions (including higher temperatures and pressure). Contemporary theoretical equations use short-range order and nonrandom molecule orientations resulting from variances in molecular size into account utilizing the excess molar Gibbs energy ($G^{ex}$) and local composition theory. The activity of a solute, for instance IMC in a saturated solution is required to be equivalent to the activity of the solute in its pure solid-state form which perform the ability of the solute solubility determination in a solution at a particular temperature. This can be done by employing a solid–liquid equilibrium (SLE) framework to apply the solute’s activity in the saturated solution, as stated in Eq. 2 [28, 29]:

2 $$\ln x_{1} = - \ln \gamma_{1} + \frac{{\Delta_{fus} H}}{R}\left( {\frac{1}{{T_{{m_{1} }} }} - \frac{1}{T}} \right) - \frac{1}{RT}\int_{{T_{{m_{1} }} }}^{T} {\Delta C_{P1} dT} + \frac{1}{R}\int_{{T_{{m_{1} }} }}^{T} {\frac{{\Delta C_{P1} }}{T}dT}$$

where *T*_*m*__1_ and *T* are the melting and experimental temperatures, respectively and R stands for the gas constant, difference in molar heat capacity between the melting and solid states of IMC, the enthalpy of fusion and activity coefficient are denoted by $\Delta C_{P1}$, $\Delta_{fus} H$, and $\gamma_{1}$ respectively. Eventually, using appropriate simplifying [30], the simplified equation obtained as:

3 $$\ln x_{1} = \frac{{\Delta_{fus} H}}{R}\left( {\frac{1}{T}_{m} - \frac{1}{T}} \right) - \ln \gamma_{1} .$$

The activity coefficient, enthalpy of fusion, and melting temperature information are required.

The experimental solubility data of IMC were correlated as a result of this project.

To generalize the e-NRTL and Wilson models for multicomponent systems including electrolytes in the aqueous solution, the molar excess Gibbs energy ($G^{ex}$) is demonstrated as the total of two contributions [31, 32]:

4 $$\frac{{G^{ex*} }}{RT} = \frac{{G^{ex*,LR} }}{RT} + \frac{{G^{ex*,SR} }}{RT},$$

where the superscripts LR, SR, and ex* denote the asymmetric convention, long-range interaction, and short-range interaction, respectively. The expanded Pitzer–Debye Hückel, G^ex*^, PDH model, which Pitzer [33] presents, could be utilized for interactions encompassing long-range terms. For short-range interactions, the Wilson and e-NRTL models were additionally applied.

### Wilson activity coefficient model

The following definitions refer to the Wilson model’s activity coefficient based on composition and temperature [17]:

5 $$\ln \gamma_{i} = 1 - \ln \left( {\sum\limits_{j = 1}^{n} {(\Lambda_{ij} x_{j} )} - \sum\limits_{k = 1}^{n} {\left( {\frac{{\Lambda_{ki} x_{k} }}{{\sum\limits_{j = 1}^{n} {(\Lambda_{kj} x_{j} )} }}} \right)} } \right),$$

where molar volumes of solute and solvents ($\upsilon$), the binary interaction parameter ($\Lambda_{ij}$) is based on characteristic energy ($\lambda$) and can be generated by Eq. 6 [34].

6 $$\Lambda_{ij} = \frac{{\upsilon_{j} }}{{\upsilon_{i} }}\exp \left( { - \frac{{\lambda_{ij} - \lambda_{ii} }}{RT}} \right).$$

### Modified Apelblat equation

The modified Apelblat equation has been utilized as the appropriate mathematical model for illustrating the substance’s polar and nonpolar behaviour. As a consequence, the calculated values were fitted with the experimental solubility [35]. Equation (7) also illustrates the solubility of IMC temperature dependence [18, 36].

7 $$\ln x_{1} = A + \frac{B}{T}C\ln T,$$

where empirical constants A, B, and C were indicated. The values of A and B reveal how the solution activity coefficient varies, while the value of C illustrates how temperature affects fusion enthalpy.

### Jouyban–Acree–Van’t Hoff model

The equation of Van’t Hoff is another model which illustrates how the natural logarithm of solubility mole fraction depends on absolute temperature [37]:

8 $$\ln x_{T} = A + \frac{B}{T}.$$

The Jouyban–Acree–Van’t Hoff model could be derived and expressed as Eq. (9) [38].

9 $$\log X_{1,T} = w_{2} \left( {A_{2} + \frac{{B_{2} }}{T}} \right) + w_{3} \left( {A_{3} + \frac{{B_{3} }}{T}} \right) + \frac{{w_{2} w_{3} }}{T}\sum\limits_{i = 0}^{2} {J_{i} (w_{2} - w_{3} )^{i} } ,$$

*A*_2_, *B*_2_, *A*_3_, *B*_3_ and *J*_i_ demonstrate the parameters of the model.

### Modified Jouyban–Acree–Apelblat model

Semi-empirical modelling is employed in the Modified Apelblat model. Utilizing this model, it is ideal to investigate the relationship between solubility and temperature [39, 40]:

10 $$\ln x_{T} = A + \frac{B}{T} + C\ln T,$$

where x_T_ is the IMC solubility mole fraction in selected mixed solvents at temperature T (K), and A, B, and C are parameters of the equation. The Modified Jouyban–Acree–Apelblat model can be generated by substituting Eq. (10) into Eq. (9) [41].

11 $$\log X_{1,T} = w_{2} \left( {A_{2} + \frac{{B_{2} }}{T} + C_{2} \ln T} \right) + w_{3} \left( {A_{3} + \frac{{B_{3} }}{T} + C_{3} \ln T} \right) + \frac{{w_{2} w_{3} }}{T}\sum\limits_{i = 0}^{2} {J_{i} (w_{2} - w_{3} )^{i} } ,$$

where* N* is the experimental points, $\ln \gamma_{i}^{\exp }$ and $\ln \gamma_{i}^{cal}$ are the experimental and calculated activity coefficients values, respectively. Furthermore, relative deviation percent (ARD%), which is represented by Eq. 17 for the models stated, can be applied to figure out the variance in solubility data between calculated and experimental data:

12 $$ARD = 100\left( {\frac{{\sum\limits_{i = 1}^{N} {\frac{{\left| {x_{i}^{\exp } - x_{i}^{cal} } \right|}}{{\left| {x_{i}^{\exp } } \right|}}} }}{N}} \right).$$

### Thermodynamic properties of dissolution

The van’t Hoff and Gibbs equations were employed for calculating the T_hm_ = 305.55 K (the mean harmonic temperature), which was derived by considering into calculate temperatures (298.15 to 313.15 K). The thermodynamic parameters of dissolution have been evaluated by employing the apparent thermodynamic functions [42]. The IMC dissolution standard molar enthalpy, $\Delta H_{so\ln }^{^\circ }$ was obtained by Eq. 13 [43–45]:

13 $$\Delta H_{so\ln }^{^\circ } = - R\left( {\frac{{\partial \ln x_{1} }}{{\partial \left( {{\raise0.7ex\hbox{$1$} \!\mathord{\left/ {\vphantom {1 T}}\right.\kern-0pt} \!\lower0.7ex\hbox{$T$}}} \right)}}} \right)_{P} ,$$

where the universal gas constant is R (8.314 J K^−1^ mol^−1^) [46] and the absolute temperature is T, the IMC mole fraction was demonstrated by x_1_ in this equation. On the other hand, the van’t Hoff plot, which is a plot of lnx_1_ versus $${\raise0.7ex\hbox{$1$} \!\mathord{\left/ {\vphantom {1 T}}\right.\kern-0pt} \!\lower0.7ex\hbox{$T$}} - {\raise0.7ex\hbox{$1$} \!\mathord{\left/ {\vphantom {1 {T_{hm} }}}\right.\kern-0pt} \!\lower0.7ex\hbox{${T_{hm} }$}}$$, can be utilized to figure out:

14 $$\Delta H_{so\ln }^{^\circ } = - R\left( {\frac{{\partial \ln x_{1} }}{{\partial \left( {{\raise0.7ex\hbox{$1$} \!\mathord{\left/ {\vphantom {1 T}}\right.\kern-0pt} \!\lower0.7ex\hbox{$T$}} - {\raise0.7ex\hbox{$1$} \!\mathord{\left/ {\vphantom {1 {T_{hm} }}}\right.\kern-0pt} \!\lower0.7ex\hbox{${T_{hm} }$}}} \right)}}} \right)_{P} ,$$

15 $$\Delta G_{so\ln }^{^\circ } = - RT_{hm} \times {\text{ intercept}}{.}$$

In this manner, the values of $\Delta H_{{{\text{soln}}}}^{^\circ }$ and $\Delta G_{{{\text{soln}}}}^{^\circ }$ have been evaluated utilizing the slope and intercept of Eqs. 14 and 15. Furthermore, the following equation [47, 48] was applied to evaluate the standard molar entropy of dissolution, $\Delta S_{{{\text{soln}}}}^{^\circ }$ [49]:

16 $$\Delta S_{so\ln }^{^\circ } = \frac{{\Delta H_{so\ln }^{^\circ } - \Delta G_{so\ln }^{^\circ } }}{{T_{hm} }}.$$

Finally, in the IMC dissolution process, Eqs. 17 and 18 were utilized to compare the relative contributions of enthalpy and entropy to the standard molar Gibbs free energy, which is demonstrated by the $\xi_{H}$ and $\xi_{TS}$, respectively [50]:

17 $$\% \xi_{H} = \frac{{\left| {\Delta H_{so\ln }^{^\circ } } \right|}}{{\left| {\Delta H_{so\ln }^{^\circ } } \right| + \left| {T\Delta S_{so\ln }^{^\circ } } \right|}} \times 100,$$

18 $$\% \xi_{TS} = \frac{{\left| {T\Delta S_{so\ln }^{^\circ } } \right|}}{{\left| {\Delta H_{so\ln }^{^\circ } } \right| + \left| {T\Delta S_{so\ln }^{^\circ } } \right|}} \times 100.$$

## Results and discussions

### Solubility results

**Fig. 3:**
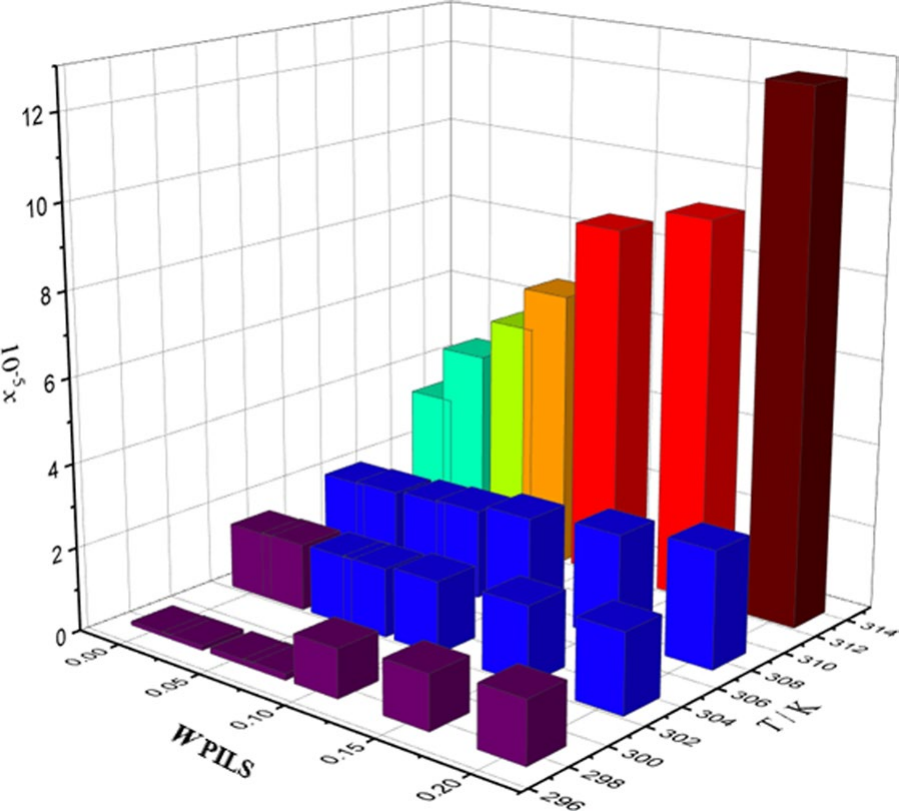
The relationship between the solubility of IMC, temperature (*T*) and weight fraction of PILs (*w*_*P*ILs_) in aqueous MEAP solutions

**Fig. 4:**
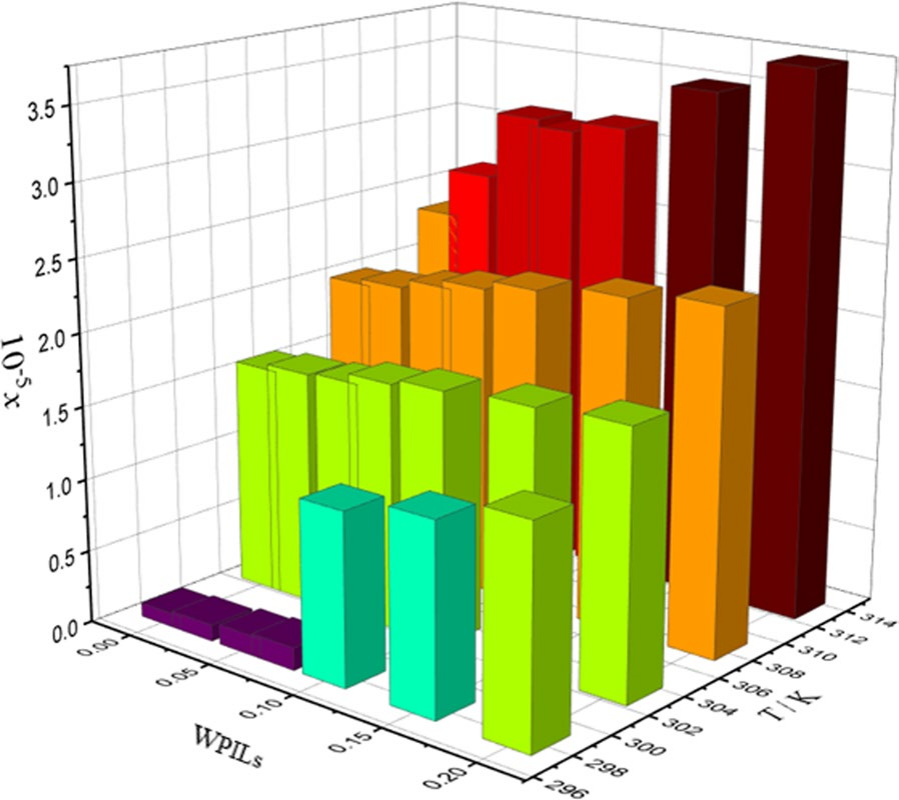
The relationship between the solubility of IMC, temperature (*T*) and weight fraction of PILs (*w*_*P*ILs_) in aqueous MEAA solutions

**Fig. 5:**
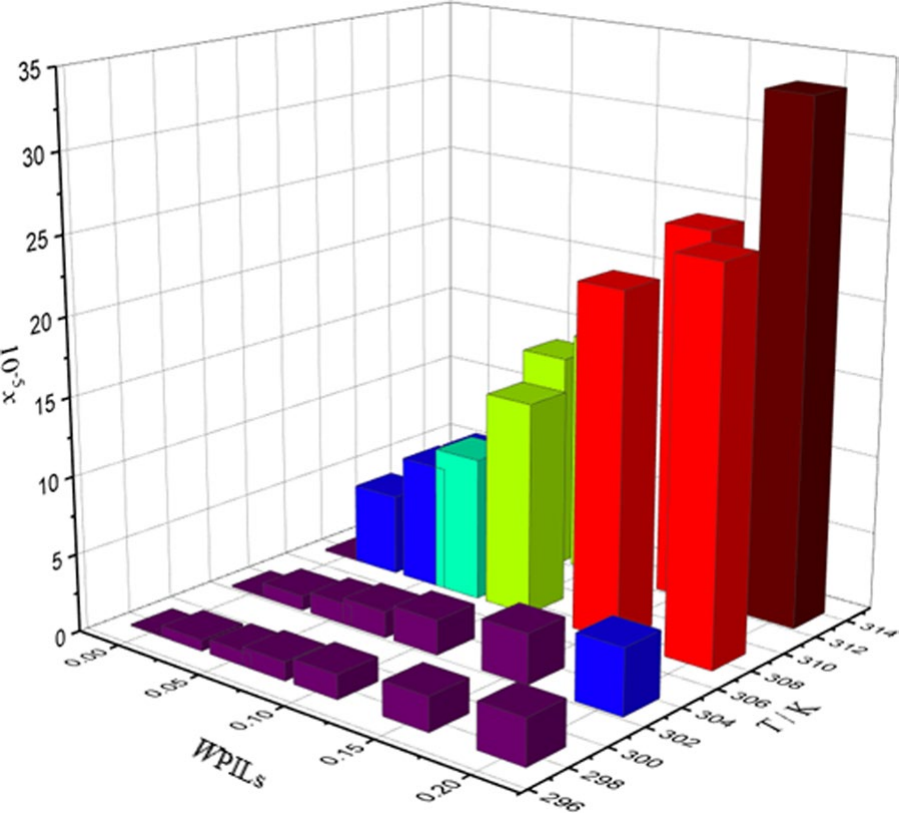
The relationship between the solubility of IMC, temperature (*T*) and weight fraction of PILs (*w*_*P*ILs_) in aqueous MEAL solutions

The indomethacin (IMC) solubility was investigated in binary solutions containing three ionic liquids: MEAP, MEAL, and MEAA. The experiments were done at various temperatures (298.15 K to 313.15 K), as considered in Table 3 and as demonstrated by Figs. 3, 4, and 5. According to the results, IMC dissolution based on experimental solubility data has enhanced as the temperature and weight fraction of systems containing protic ionic liquids have increased. Specifically, the solubility of IMC in the MEAL PIL was raised more than 200 times higher than the water at each temperature based on the evaluated experimental data from our previous investigations [26, 29].

**Table 3:** The experimental and calculated mole fraction IMC, respectively $\left( {x_{1}^{\exp } ,x_{1}^{cal} } \right)$ in different weight fractions of aqueous solution containing PILs (w_3_) calculated  from Apelblat equation and Wilson model, within the temperature range *T*/K = (298.15 to 313.15)

*T/*K	$10^{5} x_{1}^{\exp }$	Wilson model		Apelblat equation	
		$10^{5} x_{1}^{{{\text{cal}}}}$	$100\frac{{x_{1}^{\exp } - x_{1}^{cal} }}{{x_{1}^{\exp } }}$	$10^{5} x_{1}^{{{\text{cal}}}}$	$100\frac{{x_{1}^{\exp } - x_{1}^{cal} }}{{x_{1}^{\exp } }}$
Indomethacin (1) + (2-hydroxyethylammonium propionate) (2) + water (3)					
*w*_3_ = 0.0000					
298.15	0.0950	0.0970	− 0.21	0.095	0.00
303.15	0.1131	0.1131	0.00	0.114	− 0.80
308.15	0.1361	0.1388	− 1.98	0.134	1.54
313.15	0.1541	0.1548	89.95	0.155	89.94
*w*_3_ = 0.0200					
298.15	0.6977	0.7090	− 1.62	0.711	− 1.91
303.15	1.0718	0.9393	12.36	1.013	5.49
308.15	1.1721	1.1734	− 0.11	1.242	− 5.96
313.15	1.3500	1.2855	4.78	1.323	2.00
*w*_3_ = 0.0500					
298.15	1.0660	0.9183	13.86	1.06	0.56
303.15	1.3329	1.3606	− 2.08	1.34	− 0.53
308.15	1.7102	1.7183	− 0.47	1.7	0.60
313.15	2.1338	2.1194	0.67	2.14	− 0.29
*w*_3_ = 0.0700					
298.15	1.1326	1.2161	− 7.37	1.13	0.23
303.15	1.4036	1.7016	− 21.23	1.42	− 1.17
308.15	2.1169	2.1301	− 0.62	2.09	1.27
313.15	3.5159	3.4997	0.46	3.53	− 0.40
*w*_3_ = 0.1000					
298.15	1.2861	1.5763	− 22.56	1.224	4.83
303.15	1.5145	2.2448	− 48.22	1.763	− 16.41
308.15	3.3884	3.4129	− 0.72	2.9	14.41
313.15	5.1249	5.1707	− 0.89	5.402	− 5.41
*w*_3_ = 0.1500					
298.15	2.0064	2.1931	− 9.31	1.959	2.36
303.15	3.3726	3.2392	3.96	3.625	− 7.48
308.15	6.6926	6.7395	− 0.70	6.217	7.11
313.15	9.6789	9.8015	− 1.27	9.924	− 2.53
*w*_3_ = 0.2000					
298.15	3.6819	2.9278	20.48	3.72	− 1.03
303.15	6.5566	4.2401	35.33	6.365	2.92
308.15	9.2054	9.2653	− 0.65	9.494	− 3.14
313.15	12.5832	12.9659	− 3.04	12.447	1.08
Indomethacin (1) + (2-hydroxyethylammonium acetate) (2) + water (3)					
*w*_3_ = 0.0000					
298.15	0.0950	0.1130	− 18.95	0.095	0.00
303.15	0.1131	0.1360	− 20.25	0.114	− 0.80
308.15	0.1361	0.1359	0.15	0.134	1.54
313.15	0.1541	0.1541	90.00	0.155	89.94
*w*_3_ = 0.0200					
298.15	1.1127	1.1168	− 0.37	1.111	0.15
303.15	1.3036	1.3116	− 0.61	1.309	− 0.41
308.15	1.4767	1.4865	− 0.66	1.47	0.45
313.15	1.5757	1.5846	− 0.56	1.578	− 0.15
*w*_3_ = 0.0500					
298.15	1.4421	1.4547	− 0.87	1.44	0.15
303.15	1.6329	1.6124	1.26	1.64	− 0.43
308.15	1.8119	1.7601	2.86	1.81	0.10
313.15	1.9344	1.8681	3.43	1.94	− 0.29
*w*_3_ = 0.0700					
298.15	1.7277	1.6295	5.68	1.718	0.56
303.15	1.8175	1.7643	2.93	1.849	− 1.73
308.15	1.9920	1.9445	2.38	1.957	1.76
313.15	2.0279	2.0588	− 1.52	2.04	− 0.60
*w*_3_ = 0.1000					
298.15	1.8354	1.8354	− 2.99	1.829	0.35
303.15	1.9449	1.9449	− 3.47	1.962	− 0.88
308.15	2.1218	2.1218	− 6.06	2.102	0.93
313.15	2.2418	2.2418	− 6.58	2.248	− 0.28
*w*_3_ = 0.1500					
298.15	2.1677	2.3327	− 7.61	2.166	0.08
303.15	2.3308	2.4432	− 4.82	2.339	− 0.35
308.15	2.6593	2.8140	− 5.82	2.648	0.42
313.15	3.1312	2.9470	5.88	3.134	− 0.09
*w*_3_ = 0.2000					
298.15	3.0793	2.9079	5.57	3.063	0.53
303.15	3.1555	3.0177	4.37	3.208	− 1.66
308.15	3.4972	3.2507	7.05	3.437	1.72
313.15	3.7387	3.7933	− 1.46	3.761	− 0.60
Indomethacin (1) + (2-hydroxyethylammonium lactate) (2) + water (3)					
*w*_3_ = 0.0000					
298.15	0.0950	0.0952	− 0.21	0.095	0.00
303.15	0.1131	0.1130	0.09	0.114	− 0.80
308.15	0.1361	0.1365	− 0.29	0.134	1.54
313.15	0.1541	0.1540	90.01	0.155	89.94
*w*_3_ = 0.0200					
298.15	3.7276	3.7265	0.03	3.782	− 1.46
303.15	4.9543	4.9757	− 0.43	4.739	4.35
308.15	5.2114	5.0305	3.47	5.452	− 4.62
313.15	5.8803	5.8811	− 0.01	5.792	1.50
*w*_3_ = 0.0500					
298.15	5.8131	5.7872	0.45	5.937	− 2.13
303.15	7.7212	7.2932	5.54	7.231	6.35
308.15	8.0173	6.6930	16.52	8.575	− 6.96
313.15	10.1444	10.2380	− 0.92	9.916	2.25
*w*_3_ = 0.0700					
298.15	6.8162	6.8576	− 0.61	9.316	− 36.67
303.15	8.7733	9.0785	− 3.48	10.556	− 20.32
308.15	9.2110	9.2298	− 0.20	12.795	− 38.91
313.15	13.8965	13.0025	6.43	16.515	− 18.84
*w*_3_ = 0.1000					
298.15	9.4937	9.5192	− 0.27	7.006	26.20
303.15	10.9401	11.8068	− 7.92	8.059	26.34
308.15	13.7142	13.8379	− 0.90	10.044	26.76
313.15	15.6214	16.9924	− 8.78	13.494	13.62
*w*_3_ = 0.1500					
298.15	13.7739	13.8238	− 0.36	9.415	31.65
303.15	17.1638	16.5888	3.35	11.237	34.53
308.15	22.1283	21.5470	2.63	13.344	39.70
313.15	24.1457	24.2454	− 0.41	15.770	34.69
*w*_3_ = 0.2000					
298.15	20.7177	20.4932	1.08	13.640	34.16
303.15	22.5268	22.0202	2.25	17.697	21.44
308.15	24.9773	30.3173	− 21.38	21.452	14.11
313.15	33.3346	32.3623	2.92	24.399	26.81

Relative standard uncertainties: u_r_(*x*_*1*_^*exp*^) = 0.1, u*(w*_*3*_*)* = 0.0005 and u(*T*) = 0.01 K

The XRD diffractograms of raw IMC crystals together with residuals crystals sampled from solubility measurement are depicted in Figure S4. (supporting information). It should be noted that quantitative analysis of phases in samples with preferential orientation or texture is not possible using traditional methods such as direct comparison. Then the XRD patterns of the excess solid in saturated solution were compared with the IMC pattern in water which is apparent that the characteristic peaks of residual solids are identical with those of raw IMC, indicating no phase transformation occurred in solubility measurements [51]. First, the stability of IMC in the presence of PILs was investigated. The analysis of the bottom phase(s) after the solubility experiments have been presented. There are different type of factors affecting the peak intensity of XRD. These type of factors are listed as:

1. The nature of the sample under test (single-phase or multiphase of the sample).
2. Powder samples have less peak intensity than bulk samples. (Due to the effect of absorption factor).
3. Grain size: the larger the grain size, the higher the peak intensity. For example, considering samples with the same chemical composition, which have been annealed at different times of heat treatment, a sample will have a higher peak intensity with a longer heat treatment time (longer annealing time = larger grain size). It should be noted that the smaller the grain size, the higher the grain size, which is the amorphous part of the material, and as a result the background scatter in the diffraction pattern will increase and the peak intensity would decrease.

On the flip side, Limited research has been conducted on systems involving co-solvents. In a study by Peña et al. the dissolution of indomethacin in ethanol/water (wETOH = 0.2) at 298.15 K was measured to be 1.13 × 10^−5^ (mole fraction). In our investigated system, which consisted of MEA/carboxylate, the solubility of IMC was found to be 20.71 × 10^−5^ at the same weight fraction and temperature. This indicates an improvement in the solubility of IMC when using these ionic liquids. Another study by Holguín et al. [52] reported the IMC solubility mole fraction in mixtures of propylene glycol/water, with the 0.4 weight fraction for propylene glycol, as 7.61 × 10^–6^ at 303.15 K. This value is less soluble than that found in our system with MEA/carboxylate. Furthermore, the order of experimental solubility enhancement is MEAL > MEAP > MEAA. The increase in solubility can be attributed to various factors, encompassing the melting point, enthalpy of fusion, hydrogen bonding interactions, polarity, and solute–solvent interactions [53, 54]. The presence of interactions of hydrogen bonding between indomethacin, acting as the acceptor of hydrogen bond, and the MEA/carboxylic acid-based protic ionic liquids (PILs) could be the explanation for enhanced solubility of IMC in PILs-containing aqueous systems. In simpler terms, the experimental dissolution data reveal that strong hydrogen bonding interaction directly affects IMC dissolving, with propionic acid, acetic acid, and lactic acid’s acidity properties contributing to stronger hydrogen bonding interactions [54, 55]. The significant increase in IMC solubility in PIL systems indicates the presence of multiple interactions, encompasses hydrogen bonding and strong ion–dipole interactions as compared to dipole–dipole interactions [56].

### Correlation results

Subsequently, the experimental solubility data were investigated using different thermodynamic models, including the Modified Jouyban–Acree–Apelblat, Jouyban–Acree–Van’t Hoff, Modified Apelblat equation, and Wilson model as the local composition model. Tables 4, 5, 6 and 7 present the collected obtained outcomes along with their corresponding parameters. It is noteworthy that Shekaari et al. conducted DSC experiments to determine the melting point (T_m_ = 432.6 K) and enthalpy of fusion of the IMC at the previous investigations. Furthermore, Table 8 summarizes the percentages of average relative deviation (ARD%) for the correlation performance of these models. The results indicate the models performance as the ordered: Modified Apelblat–Jouyban–Acree > Van’t Hoff–Jouyban–Acree > Modified Apelblat equation > Wilson models for the aqueous solutions containing PILs. Table 7 presents the calculated activity coefficients ($\gamma_{1}^{{}}$) for IMC in these systems. It could be observed that the ($\gamma_{1}^{{}}$) values decrease with an enhancement in the weight fraction of the protic ionic liquids (PILs) present in these systems. This observation confirms that the activity coefficient decreases as a result of increased interactions [30].

**Table 4:** The modified Apelblat–Jouyban–Acree mode’s parameters for the IMC in the systems that are investigated

*T*/K	10^3^*A*_1_	10^3^ *B*_1_	*C*_1_	*A*_2_	10^4^ *B*_2_	10^3^ *C*2	10^–3^ *J*_0_	10^–4^ *J*_1_	10^–4^ *J*_2_
Indomethacin (1) + (2-hydroxyethylammonium propionate) (2) + water (3)									
298.15	0.145	− 0.625	6.745	− 10.531	− 8.307	− 292.000	0.001	5.047	4.205
303.15	26.000	24.000	1.677	− 8.267	− 1.527	− 553.000	− 0.001	0.923	0.289
308.15	− 5.640	− 5.001	− 250.491	− 11.591	− 1.996	0.014	804.900	50.640	14.401
313.15	5.406	− 13.000	− 5.573	− 11.566	− 0.488	0.029	3.958	− 2.002	− 1.358

*T*/K	*A*_1_	10^5^ *B*_1_	*C*_1_	*A*_2_	10^5^ *B*_2_	10^4^ *C*2	10^–5^ *J*_0_	10^–5^ *J*_1_	10^–4^ *J*_2_
Indomethacin (1) + (2-hydroxyethylammonium acetate) (2) + water (3)									
298.15	4.493	− 1.465	− 29.302	− 11.715	− 21.190	1.157	0.981	0.927	4.486
303.15	9.601	− 1.015	− 110.745	− 11.507	− 5.295	0.580	3.564	2.607	9.461
308.15	− 200.726	− 2.930	17.477	− 11.384	− 3.065	2.317	0.766	0.977	5.343
313.15	− 0.004	− 2.939	− 802.922	− 11.426	− 3.069	2.314	26.777	17.972	56.877

*T*/K	*A*_1_	10^5^ *B*_1_	*C*_1_	*A*_2_	10^4^ *B*_2_	10^4^ *C*2	10^–5^ *J*_0_	10^–5^ *J*_1_	10^–5^ *J*_2_
Indomethacin (1) + (2-hydroxyethylammonium lactate) (2) + water (3)									
298.15	0.155	− 1.464	− 26.216	− 10.548	− 1.805	1.167	0.863	0.696	0.305
303.15	37.455	− 1.424	− 905.832	− 10.630	− 1.685	1.133	28.960	19.600	6.321
308.15	1.538	− 1.465	− 407.755	− 10.219	− 1.559	1.158	13.220	8.627	2.636
313.15	1.419	− 1.464	− 369.190	− 10.403	− 0.838	1.159	12.450	8.689	2.971

**Table 5:** The Jouyban–Acree–Van’t Hoff mode’s parameters for the IMC in the aqueous solutions containing PILs

*T*/K	*A*_1_	10^3^*B*_1_	*A*_2_	10^3^*B*_2_	*J*_0_	10^–4^ *J*_1_	10^–4^ *J*_2_
Indomethacin (1) + (2-hydroxyethylammonium propionate) (2) + water (3)							
298.15	38.424	1.625	− 12.197	1.187	3.489	5.047	4.205
303.15	9.608	7.000	− 11.427	4.750	− 1.851	0.923	0.289
308.15	− 1416.0001	53.003	− 11.590	38.001	7.940	0.499	14.170
313.15	− 19.792	0.813	− 11.566	0.594	0.872	− 2.265	− 1.440

*T*/K	10^−3^*A*_1_	*B*_1_	*A*_2_	*B*_2_	10^–4^ *J*_0_	10^–4^ *J*_1_	10^–4^ *J*_2_
Indomethacin (1) + (2-hydroxyethylammonium acetate) (2) + water (3)							
298.15	− 0.166	0.053	− 11.714	0.038	9.993	9.371	4.508
303.15	1.194	0.006	− 11.383	0.005	− 66.690	− 42.770	− 12.430
308.15	− 0.101	0.107	− 11.383	0.077	7.663	9.768	5.343
313.15	− 0.851	0.108	− 11.193	0.077	48.710	31.940	9.754

*T*/K	*A*_1_	*B*_1_	*A*_2_	*B*_2_	10^–6^ *J*_0_	10^–5^ *J*_1_	*J*_2_
Indomethacin (1) + (2-hydroxyethylammonium lactate) (2) + water (3)							
298.15	− 54.304	0.026	− 10.540	0.019	0.034	0.342	0.191
303.15	− 4540.000	0.108	− 10.583	0.078	2.559	17.330	5.595
308.15	− 2470.002	0.107	− 10.227	0.077	1.399	9.147	2.802
313.15	− 3308.999	0.107	− 10.459	0.077	1.935	13.300	4.420

**Table 6:** The Wilson model’s parameters for the IMC in aqueous solutions containing PILs

*T*/K	10^5^ *Λ*_*wd*_	10^3^*Λ*_*dw*_	10^3^*Λ*_*Cad*_	*Λ*_*dCa*_	10^4^*Λ*_*Caw*_	10^3^*Λ*_*wCa*_
Indomethacin (1) + (2-hydroxyethylammonium propionate) (2) + water (3)						
298.15	24.910	− 0.012	6914.000	− 3.476	− 4.843	1.382
303.15	23.180	− 0.012	19,945.000	− 4.365	− 4.843	0.896
308.15	0.034	4095.000	− 0.133	0.017	− 1.211	9.031
313.15	0.103	3763.000	1.589	0.487	− 0.303	121.000

*T*/K	10^5^ *Λ*_*wd*_	10^3^*Λ*_*dw*_	*Λ*_*Cad*_	*Λ*_*dCa*_	10^4^*Λ*_*Caw*_	10^3^*Λ*_*wCa*_
Indomethacin (1) + (2-hydroxyethylammonium acetate) (2) + water (3)						
298.15	29.580	− 0.023	2.447	− 2.811	− 19.370	5.817
303.15	27.830	1.492	2.542	− 3.081	− 2.362	5.656
308.15	5.793	1299.000	0.796	− 3.235	− 1.214	4.425
313.15	9.061	762.000	1.188	− 3.269	− 1.211	4.379

*L*	10^5^ *Λ*_*wd*_	10^3^*Λ*_*dw*_	10^3^*Λ*_*Cad*_	*Λ*_*dCa*_	10^3^*Λ*_*Caw*_	10^3^*Λ*_*wCa*_
Indomethacin (1) + (2-hydroxyethylammonium lactate) (2) + water (3)						
298.15	0.537	3532.000	3.495	0.966	− 0.121	− 0.115
303.15	23.130	1.234	16,916.000	− 2.481	− 1944.000	1.935
308.15	22.040	0.612	1695.000	0.010	− 497.000	− 2.425
313.15	19.750	0.540	6658.000	− 1.644	− 125.000	2.909

*D* drug (indomethacin), *w* water, anion (Pro, Ace, Lac), *Ca* cation [2-hydroxyethylammonium]

**Table 7:** The calculated activity coefficients of IMC, ln $\gamma_{1}^{{}}$ as a function of PILs mole fraction (first column) in aqueous solutions based on Wilson model at differet tempeartures

PILs weight fraction	*T* = 298.15 K	*T* = 303.15 K	*T* = 308.15 K	*T* = 313.15 K
Indomethacin (1) + (2-hydroxyethylammonium propionate) (2) + water (3)				
0.0000	9.2901	9.3601	9.4106	9.5131
0.0200	7.2978	7.1113	7.2567	7.3428
0.0500	6.8739	6.8932	6.8789	6.885
0.0700	6.8133	6.8416	6.6656	6.3856
0.1000	6.6862	6.7655	6.1952	6.0088
0.1500	6.2415	5.9649	5.5145	5.373
0.2000	5.6344	5.3001	5.1957	5.1106
Indomethacin (1) + (2-hydroxyethylammonium acetate) (2) + water (3)				
0.0000	9.1182	9.1757	9.4106	9.5137
0.0200	6.831	6.9155	7.0257	7.1882
0.0500	6.5717	6.6902	6.8211	6.9831
0.0700	6.391	6.5831	6.7264	6.9359
0.1000	6.3305	6.5154	6.6632	6.8356
0.1500	6.1641	6.3344	6.4374	6.5015
0.2000	5.8131	6.0314	6.1635	6.3242
Indomethacin (1) + (2-hydroxyethylammonium lactate) (2) + water (3)				
0.0000	9.2901	9.3601	9.4106	9.5131
0.0200	7.2978	7.1113	7.2567	7.3428
0.0500	6.8739	6.8932	6.8789	6.885
0.0700	6.8133	6.8416	6.6656	6.3856
0.1000	6.6862	6.7655	6.1952	6.0088
0.1500	6.2415	5.9649	5.5145	5.373
0.2000	5.6344	5.3001	5.1957	5.1106

**Table 8:** The* ARD*% (average relative deviation percent) values for the IMC solubility in the aqueous solutions containing PILs for T/K = 298.15 to 313.15 from the a) Jouyban–Acree–Modified Apelblat, Jouyban–Acree–Van’t Hoff Modified and Wilson model and b) Apelblat equation

(a)			
ARD%			
*T*/K	Jouyban–Acree–Modified Apelblat	Jouyban–Acree–Van’t Hoff	Modified Wilson
Indomethacin (1) + water (2) + (2-hydroxyethylammonium propionate) (3)			
298.15	0.046	0.067	2.78
303.15	0.022	0.042	6.49
308.15	0.049	0.031	3.28
313.15	0.012	0.013	0.4058
Average	0.032	0.038	3.239
Indomethacin (1) + water (2) + (2-hydroxyethylammonium acetate) (3)			
298.15	0.001	0.005	2.780
303.15	0.001	0.001	3.550
308.15	0.003	0.007	2.490
313.15	0.002	0.002	3.300
Average	0.002	0.004	3.030
Indomethacin (1) + water (2) + (2-hydroxyethylammonium lactate) (3)			
298.15	0.008	0.008	1.600
303.15	0.001	0.011	0.760
308.15	0.011	0.011	17.600
313.15	0.009	0.009	10.700
Average	0.007	0.010	7.665

(b) Apelblat			
%ARD			
*w*_*3*_	Indomethacin (1) + water (2) + (2-hydroxyethylammonium propionate) (3)	Indomethacin (1) + water (2) + (2-hydroxyethylammonium acetate) (3)	Indomethacin (1) + water (2) + (2-hydroxyethylammonium lactate) (3)
0.0000	0.750	0.750	0.750
0.0200	2.980	0.300	3.810
0.0500	4.420	0.198	0.560
0.0700	5.750	1.140	0.912
0.1000	1.770	0.610	10.300
0.1500	2.050	0.260	4.860
0.2000	1.740	1.140	2.030
Average	2.780	0.628	3.317

Standard uncertainty u(*T*) = 0.01 K, u*(P)* = 10 hPa

### Thermodynamic properties of dissolution results

**Fig. 6:**
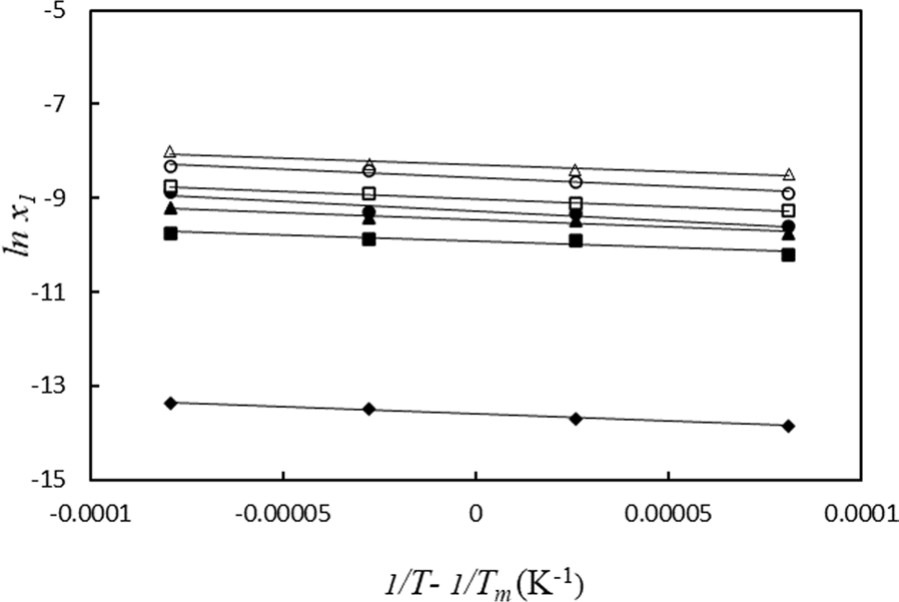
Plot of *lnx*_*1*_ vs (*1/T–1/T*_*hm*_); in aqueous solutions containing MEAL at various mass fraction of protic ionic liquids (*w*_*PILs*_): 0.0000 (black diamond suit), 0.0200 (black square), 0.0500 (black up-pointing triangle), 0.0700 (black circle), 0.1000 (white square), 0.1500 (white circle), 0.2000 (white up-pointing triangle)

**Fig. 7:**
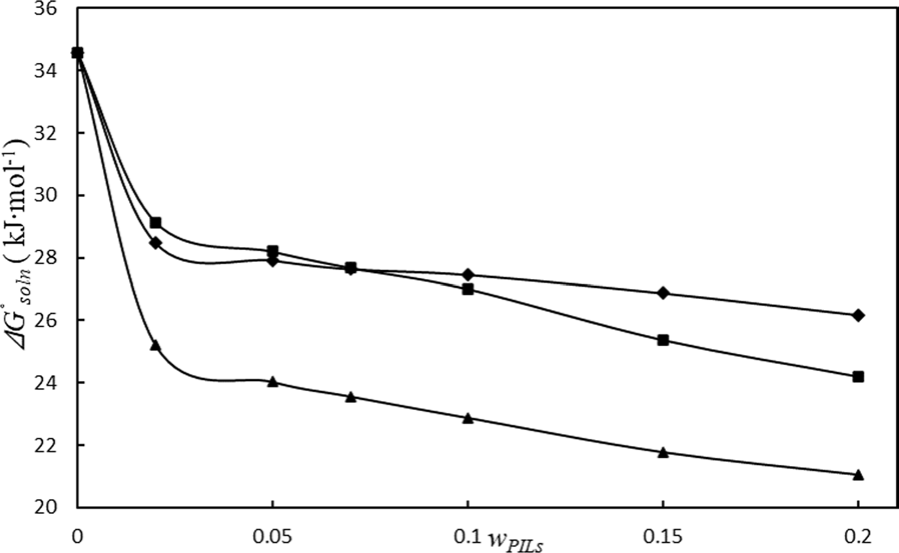
The $\Delta G_{so\ln }^{^\circ }$ values related to the process of the IMC dissolution in aqueous solutions containing PILs (MEAP (black square), MEAA (black diamond suit), MEAL (black up-pointing triangle) at 305.5 K

In the system based on the MEAL, Fig. 6. depicts the IMC solubility data ($$\ln x_{1}$$), versus $$\left( {{\raise0.7ex\hbox{$1$} \!\mathord{\left/ {\vphantom {1 T}}\right.\kern-0pt} \!\lower0.7ex\hbox{$T$}} - {\raise0.7ex\hbox{$1$} \!\mathord{\left/ {\vphantom {1 {T_{hm} }}}\right.\kern-0pt} \!\lower0.7ex\hbox{${T_{hm} }$}}} \right)$$, and Table 9 provides the amounts for the dissolution thermodynamic properties ($$\Delta H_{so\ln }^{^\circ }$$, $$T_{m} \Delta S_{so\ln }^{^\circ }$$ and $$\Delta G_{so\ln }^{^\circ }$$). The dissolution of the IMC in these systems is an endothermic process based on the positive values of ($$\Delta H_{so\ln }^{^\circ }$$) and ($$\Delta H_{so\ln }^{^\circ }$$). As can be seen in Fig. 7, the values of the standard molar Gibbs free energy ($$\Delta G_{so\ln }^{^\circ }$$) decreased as the weight fraction of PILs increased. Additionally, during the dissolution process, the ($$T_{hm} \Delta S_{so\ln }^{^\circ }$$) values have positive values.

**Table 9:** The apparent thermodynamic functions for the dissolution process at mean temperature for the IMC in aqueous PILs as a function of the PILs weight fractions (*w*_3_)

*w*_*3*_	$\Delta H_{so\ln }^{^\circ }$/Kj mol^−1^	$T_{M} \Delta S_{so\ln }^{^\circ }$/kJ mol^−1^	$\Delta G_{so\ln }^{^\circ }$/kJ mol^−1^	$\xi_{H}$	$\xi_{TS}$
Indomethacin (1) + (2-hydroxyethylammonium propionate) (2) + water (3)					
0.0000	25.32	− 9.26	34.58	73.23	26.77
0.0200	32.28	3.14	29.14	91.14	8.86
0.0500	36.19	7.99	28.20	81.92	18.08
0.0700	58.98	31.30	27.68	65.33	34.67
0.1000	76.75	49.74	27.01	60.68	39.32
0.1500	83.99	58.61	25.38	58.90	41.10
0.2000	62.63	38.42	24.21	61.98	38.02
Indomethacin (1) + (2-hydroxyethylammonium acetate) (2) + water (3)					
0.0000	25.32	− 9.26	34.58	73.23	26.77
0.0200	18.18	− 10.29	28.47	63.86	36.14
0.0500	15.32	− 12.59	27.91	54.90	45.10
0.0700	8.90	− 18.73	27.63	32.21	67.79
0.1000	10.67	− 16.78	27.45	38.86	61.14
0.1500	19.13	− 7.75	26.87	71.17	28.83
0.2000	10.61	− 15.56	26.17	40.54	59.46
Indomethacin (1) + (2-hydroxyethylammonium lactate) (2) + water (3)					
0.0000	25.32	− 9.26	34.58	73.23	26.77
0.0200	22.10	− 3.12	25.22	87.61	12.39
0.0500	26.54	2.50	24.04	91.39	8.61
0.0700	33.85	10.28	23.57	76.70	23.30
0.1000	26.70	3.81	22.89	87.50	12.50
0.1500	30.15	8.36	21.79	78.28	21.72
0.2000	23.65	2.57	21.07	90.18	9.82

Standard uncertainty of u is u(*w*_*3*_) = 0.0002 and u(*T*) = 0.01 K

According to the data, ($\Delta H_{so\ln }^{^\circ }$) has a greater impact on the dissolution of the IMC in these systems due to lower entropy values compared to enthalpy values. Based upon the Table 9, the ($\xi_{H}$) and ($\xi_{TS}$) values for the IMC's dissolving process, the $\Delta G_{so\ln }^{^\circ }$ main contribution is connected to the dissolution’s enthalpy [28].

### Cytotoxicity of PILs results

**Fig. 8:**
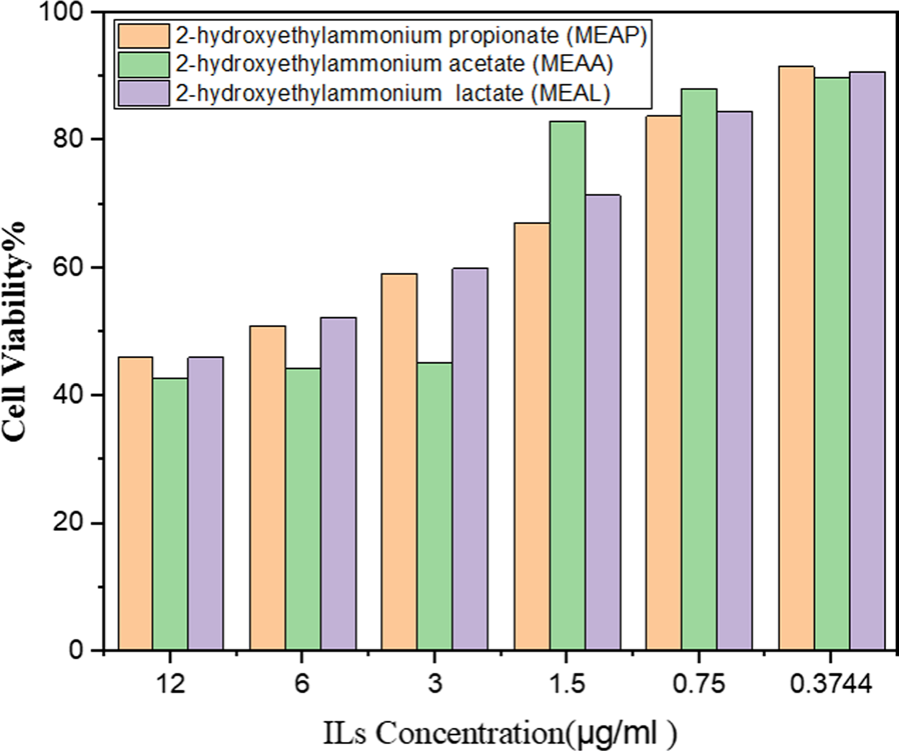
Cell viability of the human colon adenocarcinoma cell line (HT29) dose–response curves of the mentioned PILs

The MTT assay was used to investigate the effect of synthesized PILs on cell proliferation. The IC50 (half total inhibitory concentration) values were calculated using the MTT assay data. This is the compound concentration at which 50% of the cells remain viable. Table 10 and Fig. 8 demonstrate the IC_50_ findings, with the cytotoxicity order being 2-hydroxyethylammonium acetate (MEAA) > 2-hydroxyethylammonium propionate (MEAP) > 2-hydroxyethylammonium lactate (MEAL). Major differences of cytotoxicity potential exist between the contaminants. In these experimental conditions, the four categories of extracts which are; very active (IC50 ≤ 20 μg/mL), moderately active (IC50 > 20–100 μg/mL), weakly active (IC50 > 100–1000 μg/mL) and inactive (IC50 > 1000 μg/mL) [57, 58]. The cytotoxic analysis revealed that, 2-hydroxyethylammonium acetate (MEAA), 2-hydroxyethylammonium lactate (MEAL), and 2-hydroxyethylammonium propionate (MEAP) possessed moderate cytotoxic effect against the HT29 cell line with IC50 26.40 ± 0.006, 79.23 ± 0.001 and 67.19 ± 0.049 μg/mL, respectively.

**Table 10:** IC50 values for the PILs which were investigated in the HT29 cell line

PILs	IC50 (µg mL^−1^)
2-Hydroxyethylammonium acetate (MEAA)	26.40 ± 0.006
2-Hydroxyethylammonium lactate (MEAL)	79.23 ± 0.001
2-Hydroxyethylammonium propionate (MEAP)	67.19 ± 0.049

The different functional groups of MEAA, MEAL, and MEAP could influence their cytotoxicity on HT29 cells through various mechanisms. All three compounds are ionic, but the acetate, lactate, and propionate groups have different charges and polarities. These differences can affect their interactions with proteins and other biomolecules inside the cell, influencing cytotoxicity.

On the other hand, the acetate, lactate, and propionate anions can be metabolized by the cells through different pathways. These metabolic processes can affect cellular energy production, redox balance, and other vital functions, potentially leading to cytotoxicity. Additionally the specific toxicities of the functional groups could reveal the toxic effect of these compounds. Acetate, lactate, and propionate base PILs have been shown to have some intrinsic toxicity, although the mechanisms are not fully understood.

It’s noteworthy that cytotoxicity is a complex phenomenon influenced by multiple factors, and the functional groups are just one aspect. Other factors encompassing concentration, exposure time, and cell line characteristics can also play significant roles. Overall, the different functional groups of MEAA, MEAL, and MEAP likely influence their cytotoxicity on HT29 cells through a combination of mechanisms involving ionic interactions, metabolic effects, and specific toxicities of the functional groups which is demonstrating the moderately activity in this systems.

## Conclusions

In the present investigation, three protic ionic liquids 2-hydroxyethylammonium acetate (MEAA), 2-hydroxyethylammonium lactate (MEAL), and 2-hydroxyethylammonium propionate (MEAP) have been synthesized for evaluating the experimental solubility of very poorly soluble drug indomethacin (IMC). The various weight fractions of the protic ionic liquids utilized in the solubility experiments at different temperatures. The results demonstrated that increasing the temperature and weight fraction of the protic ionic liquids enhanced IMC’s solubility. Among the studied PIL, MEAL exhibited the highest solubility enhancement due to the strong hydrogen bonding interactions by the carboxylic acid and strongly hydrogenic interactions encompassing the number of hydrogen bond acceptor and donor states in acid carboxylic acid structures. Moreover, the other strong ion–dipole interactions as compared to dipole–dipole interactions has the significant role in the raising of drug solubility at the present investigation. In addition, different thermodynamic models Van’t Hoff–Jouyban–Acree, Modified Apelblat equation, and Wilson models, were applied to correlate the experimental solubility data of IMC. The performance order of these models, in terms of their correlation precision were as follows: Modified Apelblat–Jouyban–Acree > Van’t Hoff–Jouyban–Acree > Modified Apelblat equation > Wilson model. Finally, the thermodynamic dissolution process in the investigated systems was determined. The results illustrated that the enthalpy in each of the utilized PIls drives the endothermic dissolution process. On the other hand, the cytotoxicity of the PILs under study showed the order: 2-hydroxyethylammonium acetate (MEAA) > 2-hydroxyethylammonium propionate (MEAP) > 2-hydroxyethylammonium lactate (MEAL). The cytotoxicity results demonstrated that the 2-hydroxyethylammonium-based PILs have low to moderate toxicity which is influenced by encompassing concentration, exposure time, and cell line characteristics.

### Supplementary Information

Supplementary Material 1.

## Data Availability

The datasets utilized and/or analyzed during this study are available from the corresponding author on reasonable request.

## Abbreviations

VOCs: Volatile organic compounds
PILs: Protic ionic liquids
IMC: Indomethacin
MEAA: 2-Hydroxyethylammonium acetate
MEAL: 2-Hydroxyethylammonium lactate
MEAP: 2-Hydroxyethylammonium propionate
