# Supplementary material for: Effect of 2-hydroxyethylammonium carboxylate protic ionic liquids on the solubility and cytotoxicity of indomethacin
Source: BMC Chem. 2024 Jun 3;18(1):109. doi: 10.1186/s13065-024-01212-4 (PMC11145891; doi:10.1186/s13065-024-01212-4)
Supplement: Supplementary file 1 — Supplementary Material 1. [file 13065_2024_1212_MOESM1_ESM.docx]

**Effect of 2-hydroxyethylammonium carboxylate protic ionic liquids on the solubility and cytotoxicity of indomethacin**

**Parisa Akbarzadeh Gondoghdi ^a^, Mohammad Khorsandi ^a,b^, Hemayat Shekaari ^a,^**^[[1]](#footnote-1)^***, Masumeh Mokhtarpour ^a^, Hamed Hamishehkar ^b^**

^a^ Department of Physical Chemistry, University of Tabriz, Tabriz, Iran

^b^ Drug Applied Research Center, Tabriz University of Medical Sciences, Tabriz, Iran.

**
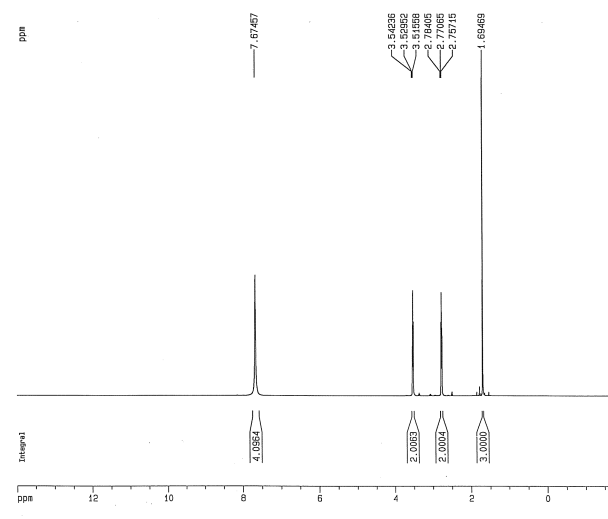
**

**Fig. S1.** ^1^H NMR of ionic liquid 2-hydroxyethylammonium acetate (400 MHz, DMSO).

**
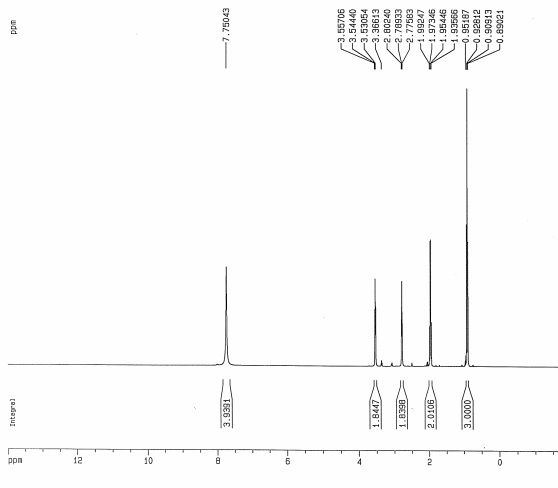
**

**Fig. S2.** ^1^H NMR of ionic liquid 2-hydroxyethylammonium propionate (400 MHz, DMSO).


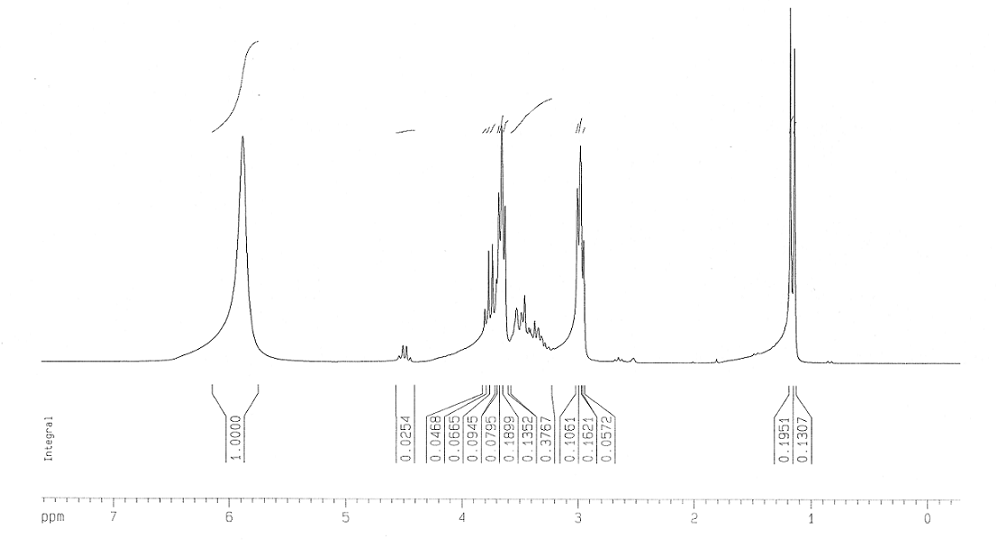


**Fig. S3.** ^1^H NMR of ionic liquid 2-hydroxyethylammonium lactate (400 MHz, DMSO).


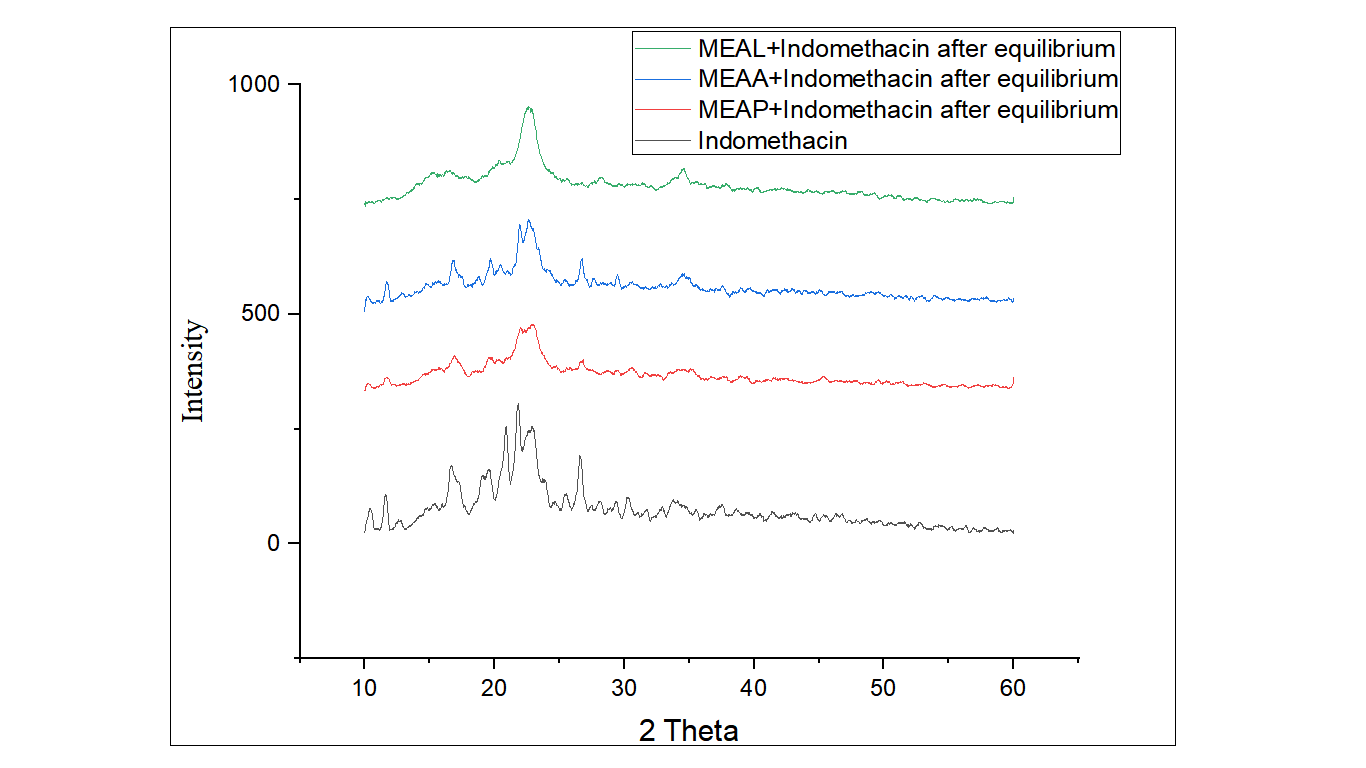


**Fig. S4.** The XRD diffractograms of raw IMC crystals together with residuals crystals sampled from solubility for 2-hydroxyethyl ammonium acetate (MEAA), 2-hydroxyethyl ammonium lactate (MEAL), and 2-hydroxyethyl ammonium propionate (MEAP).

1. * *Corresponding author. Tel.: +*98-41-33393094. Fax: +98-41-33340191.

   E-mail address: hemayatt@yahoo.com (H. Shekaari). [↑](#footnote-ref-1)
